# Supplementary figures and images for: The evolutionary mechanism of non-carbapenemase carbapenem-resistant phenotypes in Klebsiella spp
Source: eLife. 2023 Jul 6;12:e83107. doi: 10.7554/eLife.83107 (PMC10325707; doi:10.7554/eLife.83107)

## Slide 1
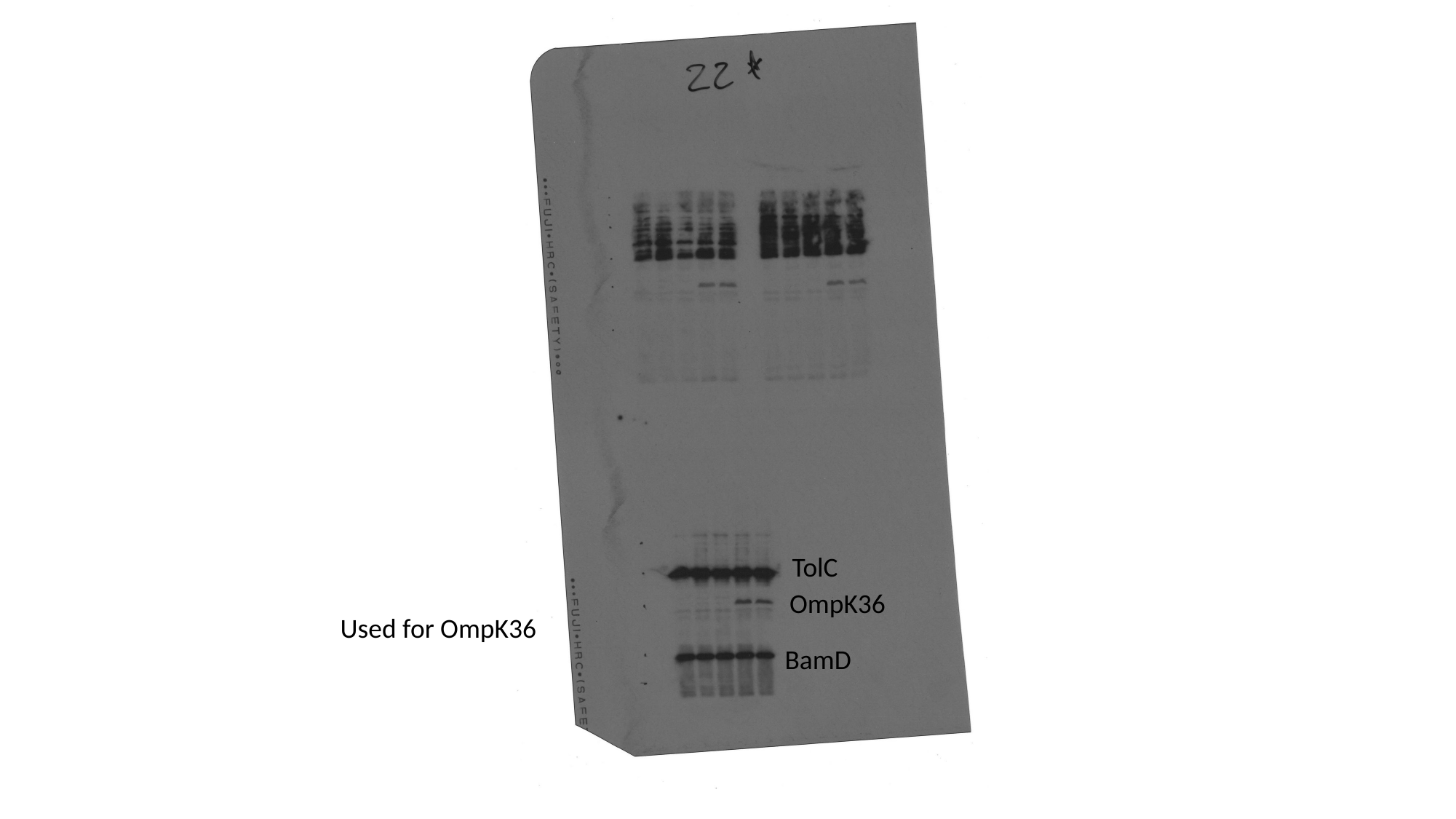

TolC
OmpK36
Used for OmpK36
BamD

Supplement: Figure 2—source data 1. [file elife-83107-fig2-data1.zip › Figure2Source1/Figure 2-figure supplement 1-source data1.pptx]

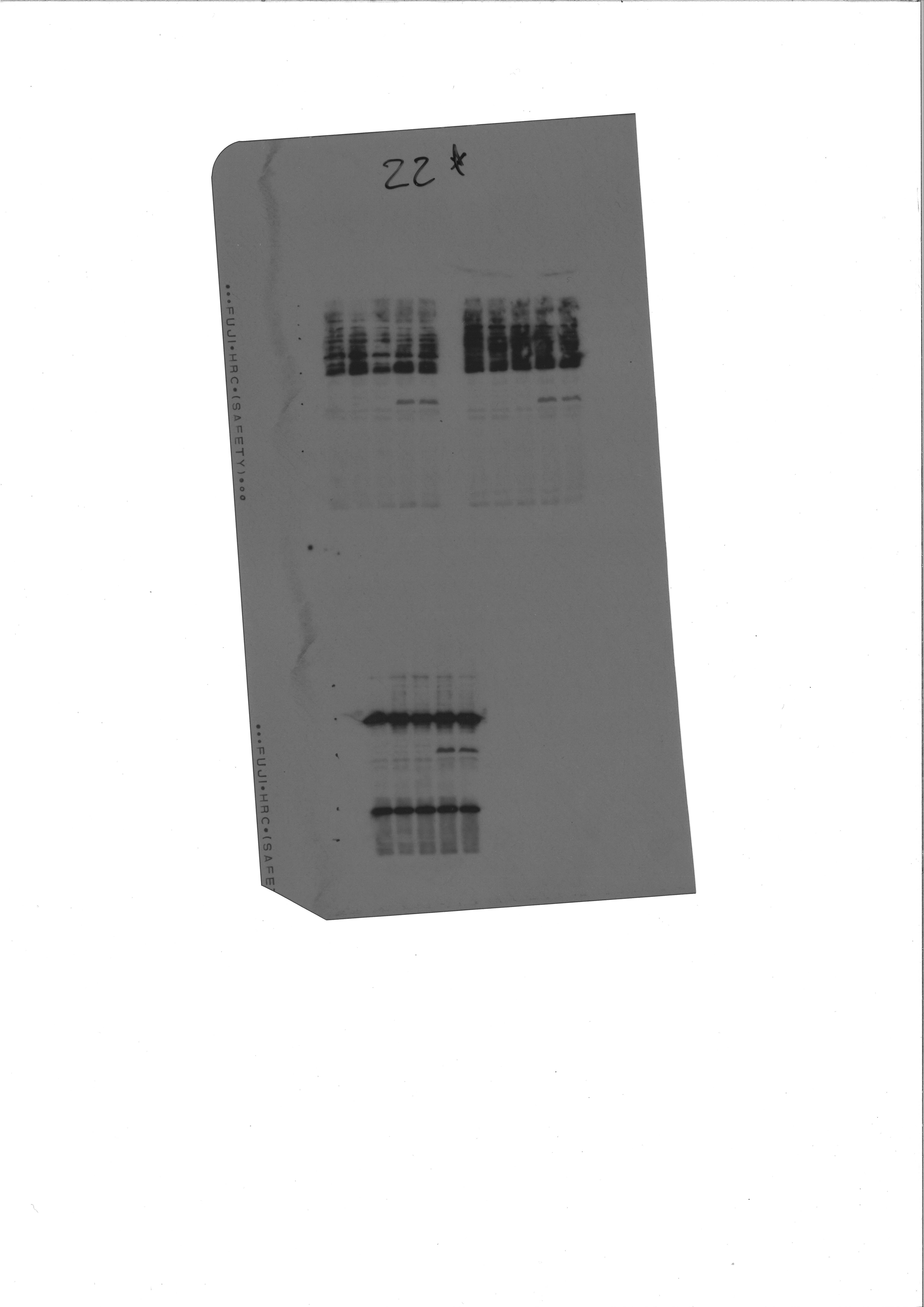

Supplement: Figure 2—source data 1. [file elife-83107-fig2-data1.zip › Figure2Source1/Figure 2-source data 1.jpg]

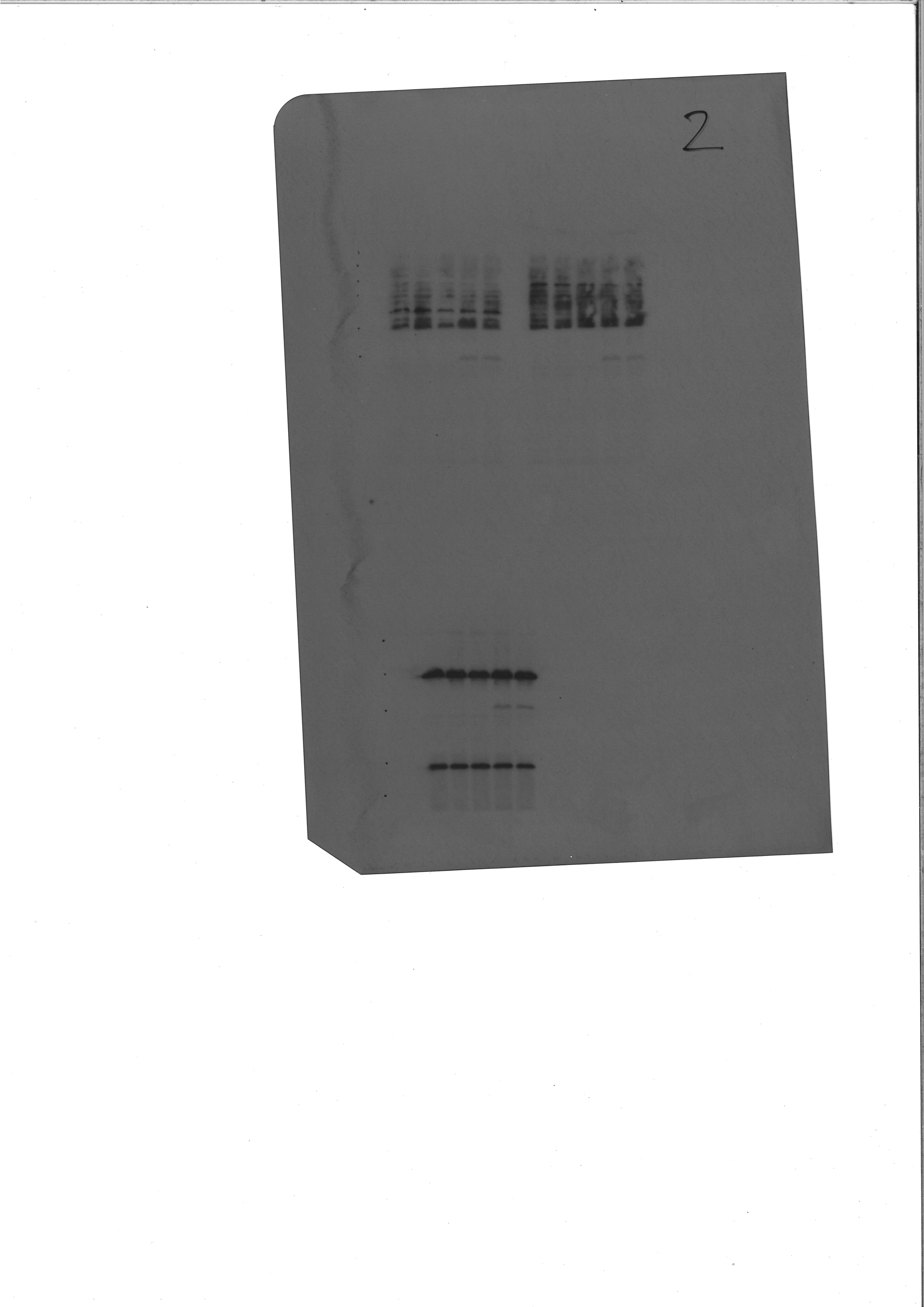

Supplement: Figure 2—source data 2. [file elife-83107-fig2-data2.zip › Figure2Source2/Figure 2-source data 2.jpg]

## Slide 1
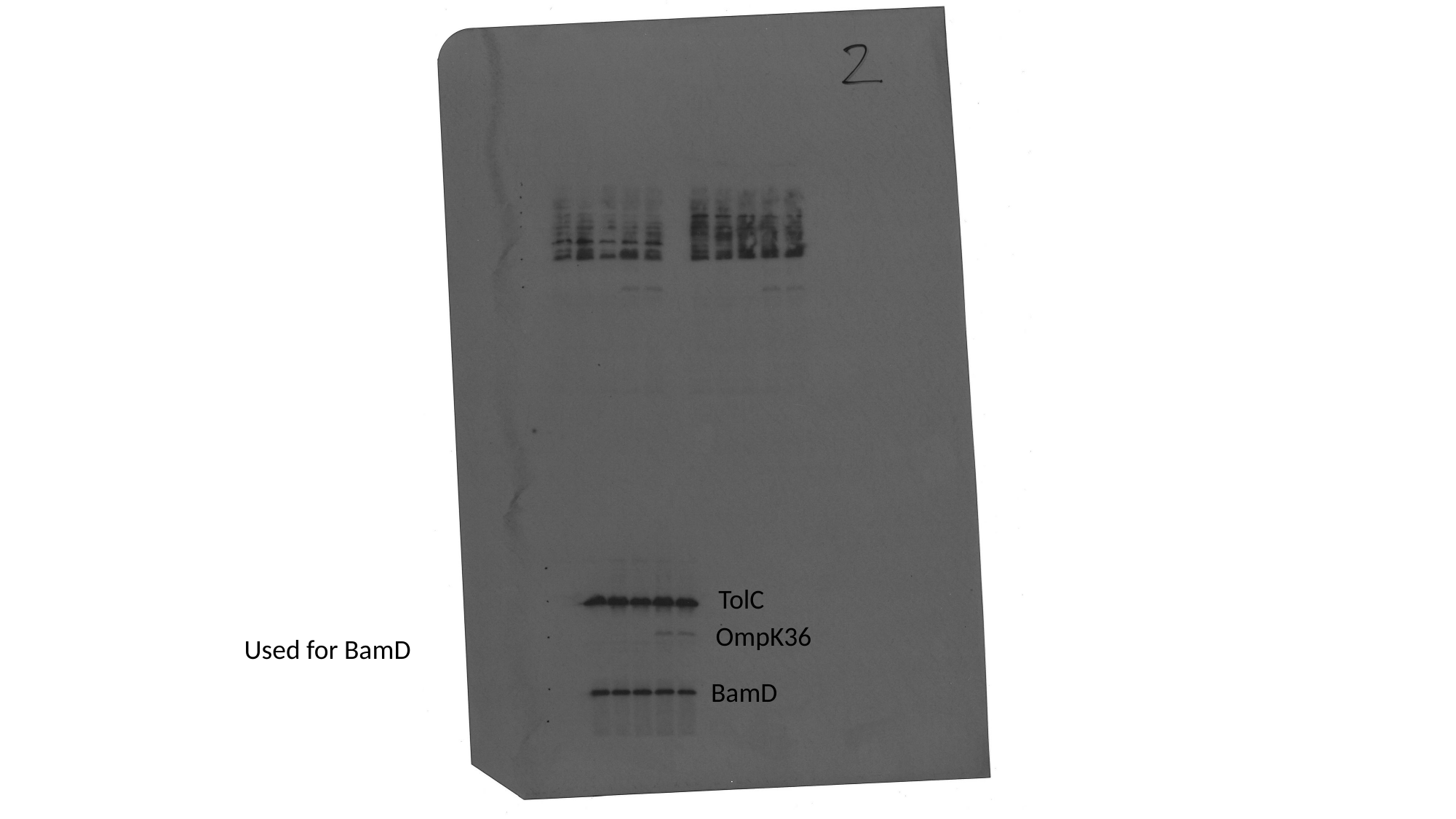

TolC
OmpK36
Used for BamD
BamD

Supplement: Figure 2—source data 2. [file elife-83107-fig2-data2.zip › Figure2Source2/Figure 2-figure supplement 1-source data2.pptx]

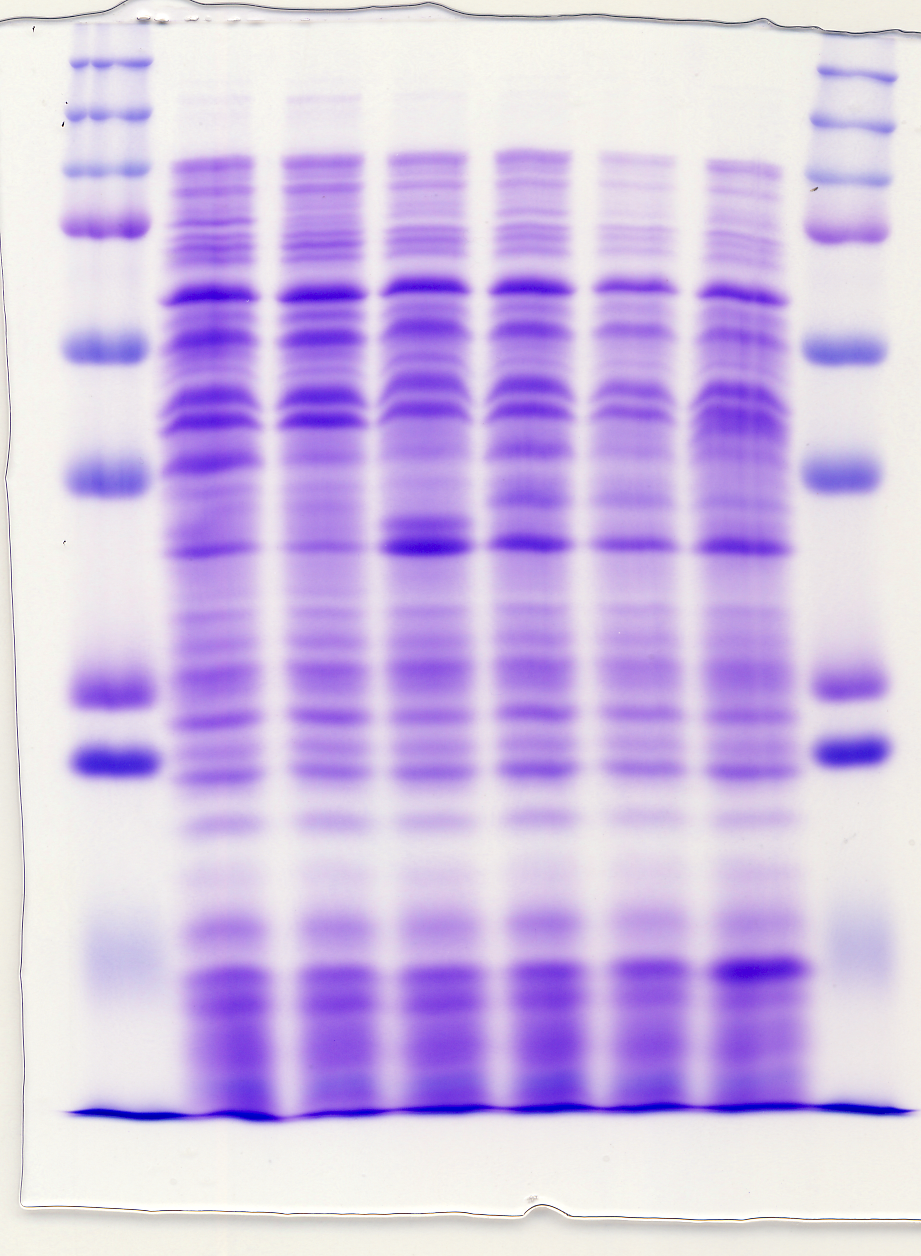

Supplement: Figure 5—source data 5. [file elife-83107-fig5-data5.zip › Figure5Source1/Figure 5-source data 1.tif]

## Slide 1
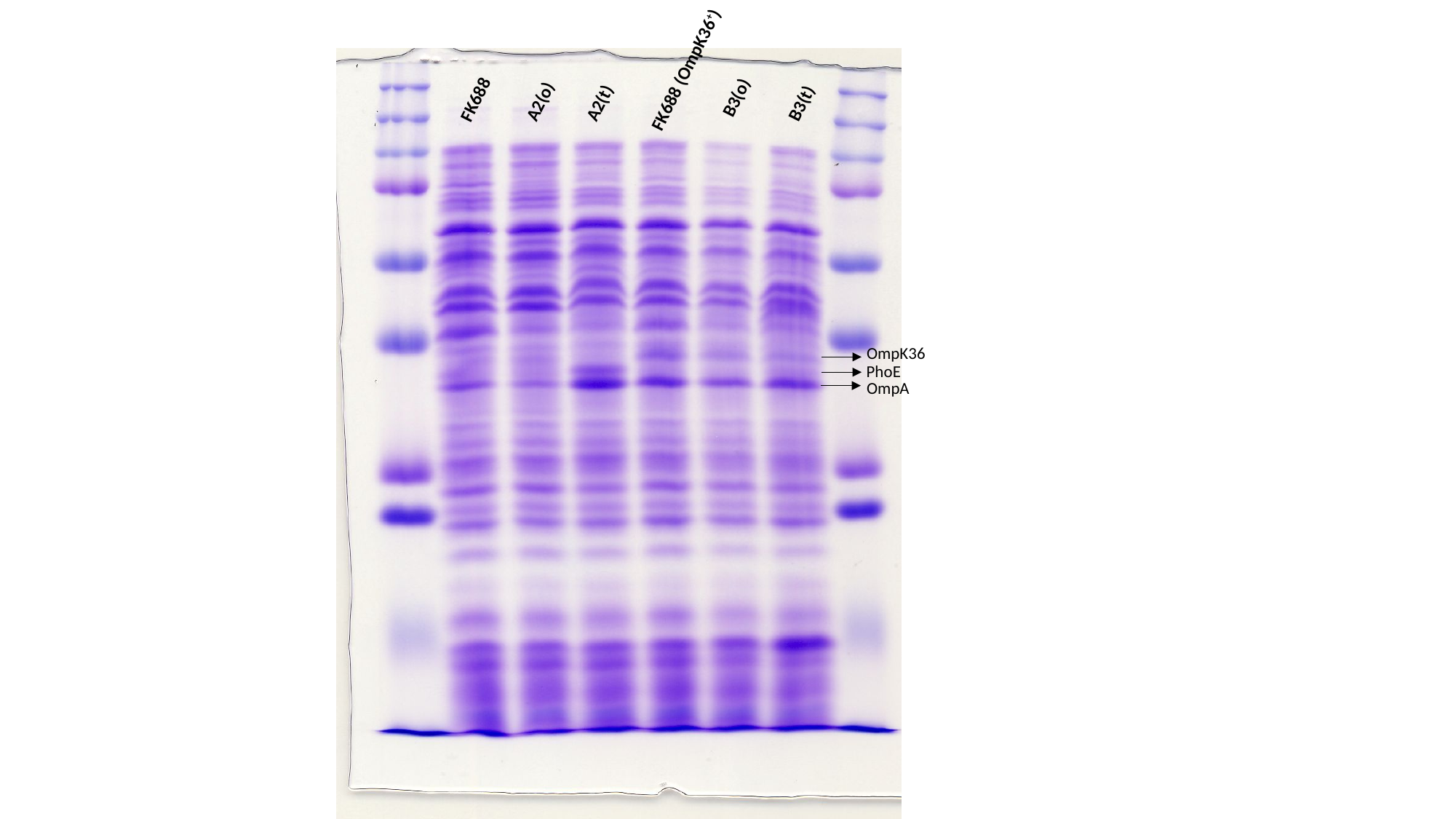

FK688 (OmpK36+)
B3(o)
FK688
A2(o)
A2(t)
B3(t)
OmpK36
PhoE
OmpA

Supplement: Figure 5—source data 5. [file elife-83107-fig5-data5.zip › Figure5Source1/Figure 5-figure supplement 1-source data 1.pptx]
